# Successful Outcome in a Middle-Aged Woman With Secondary Infertility Using Donor Oocyte In Vitro Fertilization: A Case Report From a Rural Infertility Clinic

**DOI:** 10.7759/cureus.27710

**Published:** 2022-08-05

**Authors:** Jarul Shrivastava, Akash More, Virul Shrivastava, Deepti Shrivastava

**Affiliations:** 1 Medical School, Datta Meghe Institute of Medical Sciences, Wardha, IND; 2 Anatomy, Datta Meghe Institute of Medical Sciences, Wardha, IND; 3 Obstetrics and Gynecology, Datta Meghe Institute of Medical Sciences, Wardha, IND; 4 Department of Obstetrics and Gynecology, Jawaharlal Nehru Medical College, Datta Meghe Institute of Medical Sciences (Deemed to be University), Wardha, IND

**Keywords:** in vitro fertilization (ivf), intracytoplasmic sperm injection (icsi), low amh, hypothyroidism, asherman’s syndrome, secondary infertility

## Abstract

We present the case of a 42-year-old (gravida 2, abortion 2 (G2A2)) infertile woman who visited an infertility clinic with her husband (aged 42 years). She had been married for eight years and had two abortions during this time. After the second abortion, dilatation and curettage (D&C) was done at her hometown hospital. This is a case of secondary infertility. Before coming to our center, she visited another center for infertility treatment, where in vitro fertilization (IVF) was done using self-oocyte, but intracytoplasmic sperm injection (ICSI) was not done. Unfortunately, the treatment did not succeed. On hysteroscopy, adhesions were found in the cavity, which were possibly formed due to the D&C procedure performed for past abortions. This condition is known as Asherman’s syndrome. Hence, hystero-adhesiolysis was done on January 20, 2021. Her latest report showed an anti-Mullerian hormone (AMH) value of 0.252 ng/dL, which was very low. Low AMH was indicative of low ovarian reserve. Her follicle-stimulating hormone (FSH) was 23 mIU/mL, which was too high, suggestive of poor egg quality. The antral follicular count was assessed through transvaginal ultrasound; the total count was three (one on the right, and two on the left). Ovum pick-up was done on March 15, 2021. Only one oocyte was retrieved from the patient (MI grade). The husband’s sperm count was 12 million/mL with low motility, which is known as oligoasthenozoospermia; hence, ICSI was performed. However, the embryo could not be formed. Therefore, on April 1, 2021, oocytes were retrieved from the donor, and ICSI was done using the semen sample of the husband. On April 6, 2021, two embryos at the day six stage were transferred to the patient’s uterus. Before the embryo transfer, tablet estrogen 2 mg was administered thrice daily. This was started from day two of menses till day 14th. After 14 days, the patient was given a progesterone injection for six days daily. After embryo transfer, tablet estrogen 2 mg was given thrice daily, and tablet progesterone 2 mg twice daily was given as support. After 14 days of embryo transfer, a beta-human chorionic gonadotrophic hormone (βhCG) test was done. Her βhCG was positive, and the couple was delighted. Diagnostic challenges occurred due to previous abortions, previous failure of IVF, history of hypothyroidism, AMH value of 0.252 ng/dL, and presence of adhesions. This is a case of secondary infertility with cavity adhesions with low AMH and low antral follicular count. The outcome was favorable at our fertility clinic. Because the patient is a known case of hypothyroidism, levothyroxine tablets were also prescribed.

## Introduction

When a couple cannot achieve pregnancy after one year or more of unprotected coitus, the couple is considered “infertile” [1]. For many couples, infertility is a heartbreaking tragedy leading to severe physical, social, mental, and sexual problems in their lives [2]. The World Health Organization (WHO) defines primary infertility as a condition when a woman has never gotten pregnant before, and secondary infertility as the failure to achieve pregnancy after one successful conception in the past. There can be multiple causes of infertility related to either male or female partner or both partners. Female infertility may be due to one or more reasons, such as hormonal imbalance, thyroid imbalance [3], polycystic ovary syndrome, Asherman’s syndrome [4], genital tract infections, endometriosis, fallopian tube obstruction, and congenital uterine disorders.

Fallopian tube adhesions are one of the leading causes of secondary infertility in underdeveloped nations. Fritsch initially reported intrauterine adhesions in 1894, and Asherman examined them further [4,5]. They are commonly seen as a result of damage to the endometrium in curettage, such as incomplete abortion/miscarriage (33%), postpartum hemorrhage (37%), and elective termination of pregnancy (8%). Amenorrhea, infertility, or repeated abortions are common symptoms of Asherman’s syndrome [6]. Asherman’s syndrome is most commonly associated with dilation and curettage (D&C) procedures used to end a pregnancy, treat a missed abortion or partial miscarriage, or remove a left placenta after delivery. Bleeding may occur sometimes [4].

The main causes leading to abortion can be either fetal or parental. Fetal factors include chromosomal anomalies and teratogenic or mutagenic factors such as alcohol, smoking, and drugs. Maternal causes include increased maternal age, infections, drugs, nutrition, occupational and environmental factors, uterine defects, hormonal imbalance, and endocrine disorders such as diabetes mellitus and thyroid disorders [7]. Women with hypothyroidism have diminished fertility, and if they get pregnant, they have a high risk of abortion [8]. Hypothyroidism is associated with infertility. There is increased thyrotropin-releasing hormone (TRH) production and prolactinoma, which cause a delayed response of luteinizing hormone and insufficient corpus luteum formation.

Hypothyroidism affects 2-4% of women in their reproductive years. Any woman attempting to conceive should have her thyroid evaluated, especially if there is a family history of thyroid disease, an irregular menstrual cycle, and/or a history of more than two miscarriages. Triiodothyronine (T3), thyroxine (T4), thyroid-stimulating hormone (TSH), autoimmune thyroid tests, and thyroid-stimulating immunoglobulin (TSI) tests should be included in a complete thyroid evaluation. Thyroid antibodies increase the risk of recurrent abortions in women with otherwise normal thyroid function. Autoimmune thyroid testing can be done during routine fertility workups [9].

## Case presentation

Patient information

A 42-year-old (gravida 2, abortion 2 (G2A2)) woman visited the Wardha test tube baby center with her husband (aged 42 years) with the problem of infertility. She had been married for eight years and had two abortions during this time. Thus, this is a case of secondary infertility. The couple were struggling to have a baby for the past seven years, having suffered two abortions. After her second abortion, dilatation and curettage (D&C) were done at another center. Uterine adhesions were formed as a result of the D&C. Before coming to our center, she went to other centers for treatment, but, unfortunately, those attempts did not succeed. In our center, first, hystero-adhesiolysis was done for cavity adhesions on January 20, 2021. Her recent report showed an anti-Mullerian hormone (AMH) value of 0.252 ng/dL, which was very low. The antral follicular count was assessed through transvaginal ultrasound; the total number was three (one on the right, and two on the left). Ovum pick-up of the patient was done on March 15, 2021. Only one oocyte was retrieved from the patient (MI grade). The husband’s sperm count was 12 million/mL with low motility. This is known as oligoasthenozoospermia. Because intracytoplasmic sperm injection (ICSI) was not done at the previous center and they did not have a successful outcome, we decided to perform ICSI; however, an embryo could not be formed. Subsequently, we decided to opt for the donor oocytes. On April 1, 2021, oocytes were retrieved from the donor; five oocytes were retrieved. ICSI was done using the patient’s husband, and two embryos were formed. Two embryos at day six stage were subsequently transferred.

Medical History

The patient was diagnosed with hypothyroidism seven years ago, for which she had been prescribed a 25 µg levothyroxine tablet once a day. In the past, she had suffered two miscarriages. On hysteroscopy, cavity adhesions were seen, which were later treated with hystero-adhesiolysis on January 20, 2021. There was no history of hypertension, diabetes mellitus, tuberculosis, asthma, or seizure. The husband also had no such conditions. There was no history of any mental illness.

Treatment History

In the past, D&C was done after her second abortion. The couple had taken treatment for conceiving a baby at various other centers where they used patients’ oocytes and IVF was done but no embryos were formed.

Clinical findings

General Examination

**Figure 1:**
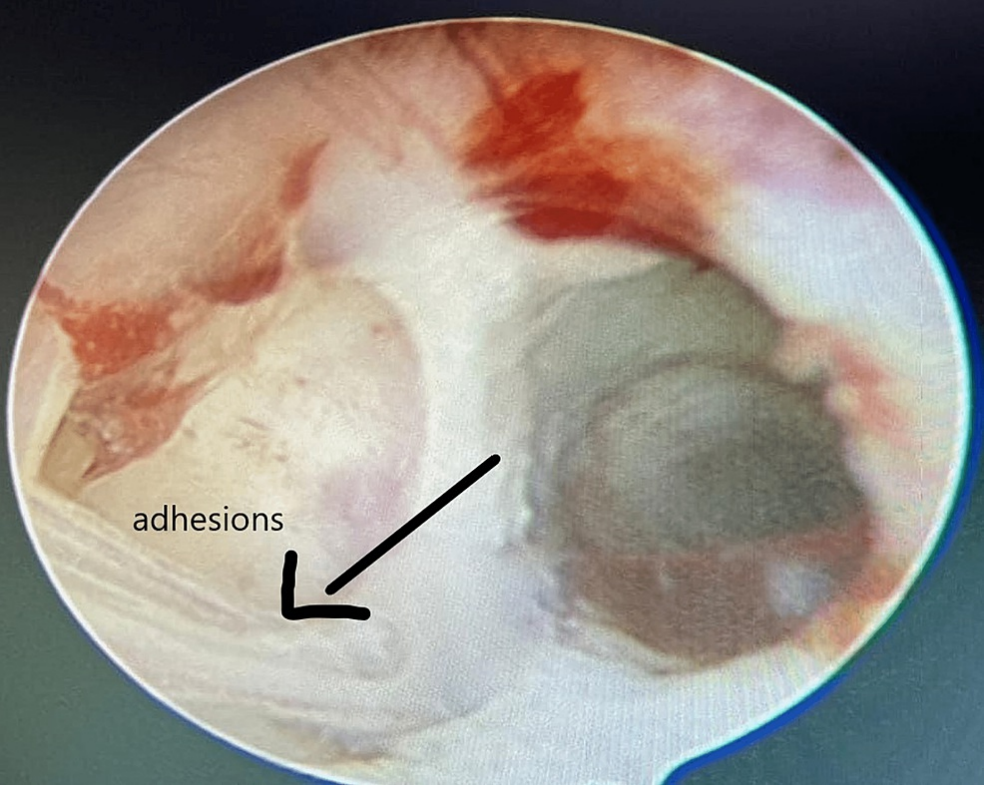
Uterine cavity showing adhesions on hysteroscopy.

On examination, all general parameters were normal. Hysteroscopy was done for the patient; the uterus was present, anteverted and anti-flexed, and cavity adhesions were seen (Figure 1). The couple underwent karyotyping, which was normal. Papanicolaou’s test of the patient was also normal.

Diagnostic Findings

**Figure 2:**
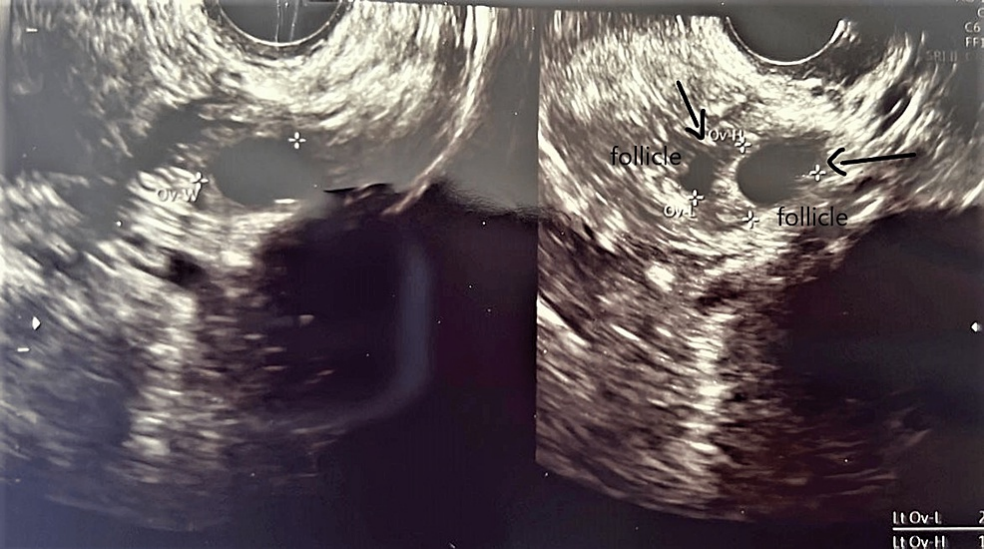
Left ovary showing two follicles.

**Figure 3:**
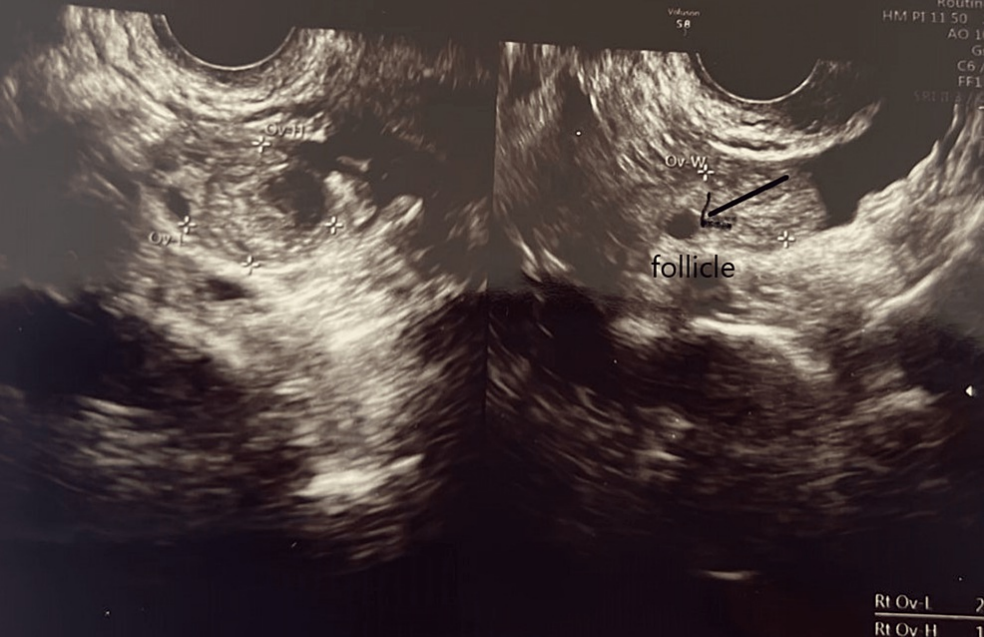
Right ovary showing one follicle.

The complete blood count of the patient was regular. The patient’s T3 and TSH levels were 1.54 µg/dL and 2.16 µg/dL, respectively, due to her ongoing treatment for hypothyroidism. AMH was 0.252 ng/dL, which was very low. The antral follicular count was assessed through transvaginal ultrasound. The total number of follicles was three, two on the left (Figure 2) and one on the right (Figure 3). FSH was 23 mIU/mL, which was too high. suggestive of poor egg quality. On ovum retrieval, only one oocyte was retrieved of MI (Metaphase I) grade, and no embryos were formed. Hence, donor oocytes were taken. The husband’s semen was utilized to prepare the embryos. ICSI was done because the husband had oligoasthenozoospermia (sperm count 12 million/mL) with low motility. Moreover, at previous centers, ICSI was not done, so we opted for ICSI. Two embryos were formed, which were transferred at the day six stage.

Diagnostic Challenges

The patient had many factors contributing to infertility. First, the patient had low AMH, high FSH, and low antral follicular count. She was a known case of hypothyroidism, which might have contributed to her abortions and secondary infertility. On hysteroscopy, uterine adhesions were seen; these adhesions were likely due to past D&C procedures done after her second abortion.

Diagnosis and Prognosis

Secondary infertility with diminished ovarian follicle and prognosis was good because adhesiolysis was performed first, followed by ICSI using the donor’s oocytes fertilized with the husband’s semen sample. Then, embryos were transferred to the patient’s uterus on day six. On day 14, beta human chorionic gonadotropin (βhCG) was positive.

Therapeutic intervention

**Figure 4:**
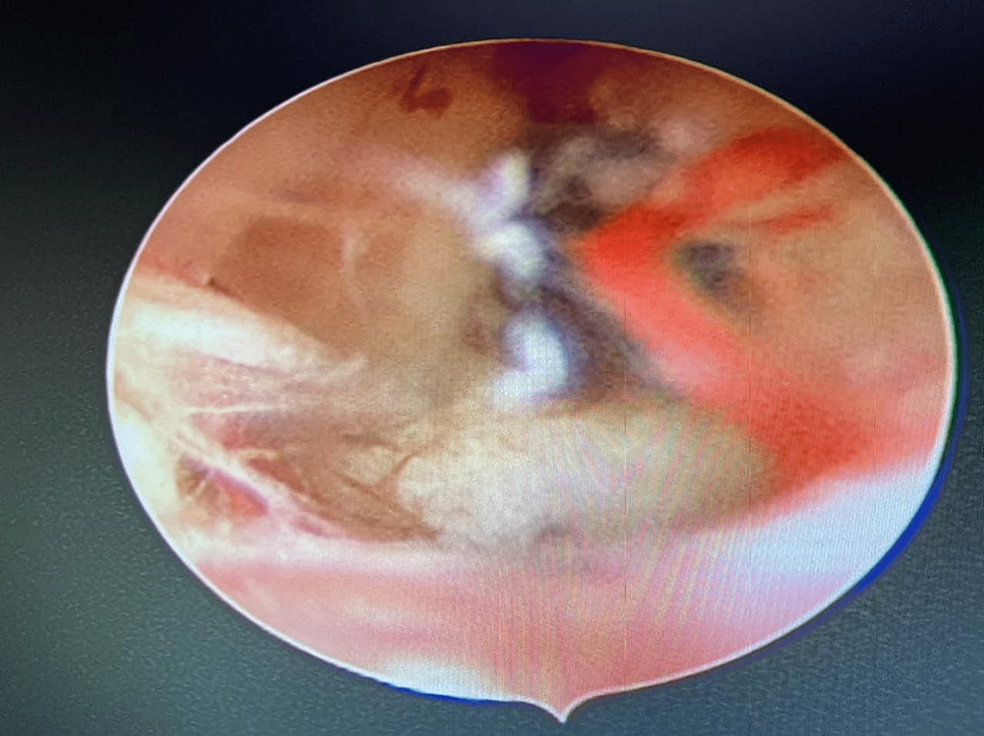
Adhesiolysis done using a 5-Fr scissor.

**Figure 5:**
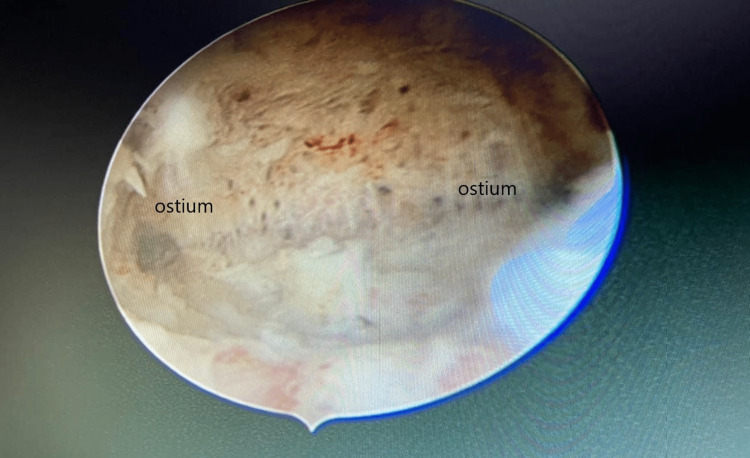
Both the ostia are clearly visible after adhesiolysis.

First, adhesiolysis was done for the adhesions on January 20, 2021 (Figures 4, 5). Because embryos could not be formed using self-oocyte, the donor’s oocytes were used with the husband’s sperm sample. In addition, ICSI was done. The embryos were then transferred on April 6, 2021. Before the embryo transfer, a tablet of estrogen 2 mg was administered thrice daily. This was started from day two of menses till day 14th. After 14 days, the patient was given a progesterone injection for six days daily. After embryo transfer, tablet estrogen 2 mg was given thrice daily, and tablet progesterone 2 mg twice daily was given for support. After 14 days of embryo transfer, a βhCG test was done. Her βhCG test was positive. The patient had been taking tablet levothyroxine 25 µg once daily for hypothyroidism.

Timeline

The couple came to the infertility clinic complaining of an inability to conceive a child after suffering two abortions during eight years of marriage. First, adhesions were removed on January 20, 2021. Then, the patient’s ovum pick-up was done, and only one MI-grade oocyte was retrieved. No embryos were formed after ICSI. Hence, oocytes were retrieved from the donor’s ovaries, and ICSI was performed using the husband’s sperm sample. As a result, two embryos were formed. On day six, two embryos were transferred. Fourteen days later, βhCG was positive.

Follow-up and outcome

Follow-up is essential after embryo transfer. After embryo transfer, tablet estrogen 2 mg was given thrice daily, and tablet progesterone 2 mg was given twice daily to support the patient. During these follow-ups, endometrium thickness was evaluated. On the 14th day, a βhCG test was done, which was positive.

## Discussion

Assisted reproductive technology (ART) is becoming more significant nowadays for couples who want to conceive. Technology has gained a significant place in the infertility management of couples. With optimism, calmness, hope, and the right treatment choice, couples can experience the joys of parenthood. About 30% of infertile couples have secondary infertility. Ectopic pregnancy, pregnancy termination, or miscarriages cause secondary infertility in different ways [10]. The history of gynecological surgeries is correlated with secondary infertility. D&C done after miscarriages can cause adhesion/synechia in the uterine cavity, a condition known as Asherman’s syndrome [6].

Even if the woman conceives after treatment of Asherman’s syndrome, strict surveillance should be done due to the possibility of placental anomalies. Adhesiolysis is a critical choice in the management of Asherman’s syndrome. Hysteroscopy has visibly improved the fertility result and success rate. Nevertheless, recurrence rates of Asherman’s syndrome are still very high, and we should keep looking for techniques that decrease the formation of adhesions [6,11].

A study by the American college of obstetricians and gynecologists suggested that AMH levels decrease after ovarian surgery, thus reducing ovarian reserve. In general, fertility reduces with age (after age 32) [12]. It is also known that levels of AMH decrease with age, thus reducing the number of oocytes available for fertilization. In such cases, it is sensible to use donor oocytes for ICSI. Success rates after using donor oocyte treatment are relatively good for the receiver above 40 years of age [12,13]. One report published in the Journal of Assisted Reproduction and Genetics by Kavoussi et al. illustrated the case of a woman who received IVF treatment and was now an oocyte donor for an infertile couple. She had a great reaction to ovarian stimulation. The numbers of oocytes retrieved were good and led to the formation of good quality of day six embryos, which is the embryo transfer cycle, resulting in a successful pregnancy for an infertile couple (recipient) [14,15]. This study showed the first such case of using donor oocytes in an ICSI for an infertile patient, where the donor herself conceived via IVF [14]. This also proves that fertility potential is good in women who have undergone IVF and can be good oocyte donors. This is the first successful report of a pregnancy where an oocyte donor for ICSI herself conceived via IVF [14]. The oocyte donor’s ovarian reserve is the main criterion for choosing oocytes, and AMH is a good predictor of ovarian response [16].

Patients with a history of one or two spontaneous miscarriages affect the fertility rate and the live birth rate in the first IVF cycle. In such women, there is a significant increase in the abortion rate, with a decrease in live birth rates, suggesting the causes of miscarriage should be found sooner [17].

Spontaneous abortion commonly occurs in advanced maternal age, in patients with a history of previous abortions, smoking, usage of certain drugs (e.g., alcohol), any chronic diseases, and endocrinal disorders (e.g., diabetes, hypertension, thyroid disorders) in the mother.

This patient had suffered two abortions in the past. Additionally, her age of marriage was late (above 32), so advanced maternal age can also contribute to previous abortion, secondary infertility, and low AMH. D&C done after abortions formed adhesions in the uterine cavity, which further impaired fertility. Thus, adhesiolysis, using donor oocytes, followed by ICSI was the most suitable option for her, which fortunately resulted in a successful outcome. The couple was glad when they could conceive. ART gave them the utmost happiness.

## Conclusions

ART has helped a large percentage of infertile couples to conceive. Late marriage and pregnancy contribute to spontaneous abortions and primary and secondary infertility. Associated health issues, such as hypothyroidism, make it harder for the woman to conceive as hypothyroidism is often correlated with infertility or miscarriages.

The D&C procedure was done after the second abortion which could be one of the reasons for the uterine adhesions (Asherman’s syndrome). Such conditions call for proper therapeutic (thyroid treatment) and surgical interventions (adhesiolysis), followed by suitable ART. Low AMH and high FSH indicate poor oocyte reserve and quality, and thus chances of embryos being formed are fewer. In such cases, it is sensible to opt for donor oocytes with the patient’s consent. Low sperm count and low sperm motility may hinder the IVF process so ICSI was done in our case. In previous centers, ICSI was not done, and a successful outcome did not occur, so we avoided that risk and chose ICSI.

Our case study may not suggest just one cause of secondary infertility because there are many contributing factors such as hypothyroidism, previous abortions, and advanced maternal age. Our case study may not surely indicate why the patient’s ART failed at other centers but was successful at our fertility clinic. There is a possibility that the successful results at this center were due to the use of donor oocytes and ICSI.
